# Stochastic simulation of successive waves of COVID-19 in the province of Barcelona

**DOI:** 10.1016/j.idm.2022.12.005

**Published:** 2022-12-27

**Authors:** M. Bosman, A. Esteve, L. Gabbanelli, X. Jordan, A. López-Gay, M. Manera, M. Martínez, P. Masjuan, Ll.M. Mir, J. Paradells, A. Pignatelli, I. Riu, V. Vitagliano

**Affiliations:** a Institut de Física d’Altes Energies (IFAE), The Barcelona Institute of Science and Technology, Barcelona, Spain; b Centre d’Estudis Demogràfics (CED-CERCA), Barcelona, Spain; c Serra Húnter Fellow, Departament de Ciències Polítiques i Socials, Universitat Pompeu Fabra, Barcelona, Spain; d i2CAT Foundation, Edifici Nexus (Campus Nord UPC), Barcelona, Spain; e Departament de Geografia, Universitat Autònoma de Barcelona, Bellaterra, Spain; f Serra Húnter Fellow, Departament de Física, Universitat Autònoma de Barcelona, Bellaterra, Spain; g Institució Catalana de Recerca i Estudis Avançats (ICREA), Barcelona, Spain; h Departament de Física, Universitat Autònoma de Barcelona, Bellaterra, Spain; i Departament d’Enginyeria Telemàtica, Universitat Politècnica de Catalunya, Barcelona, Spain; j DIME, University of Genova, Via all’Opera Pia 15, 16145, Genova, Italy; k INFN, Sezione di Genova, via Dodecaneso 33, 16146, Genoa, Italy; l Department of Mathematics and Physics, University of Hull, Kingston upon Hull, HU6 7RX, UK

**Keywords:** COVID-19 modelling, Parameter estimation, Socio-demographic data, Intervention

## Abstract

Analytic compartmental models are currently used in mathematical epidemiology to forecast the COVID-19 pandemic evolution and explore the impact of mitigation strategies. In general, such models treat the population as a single entity, losing the social, cultural and economical specificities. We present a network model that uses socio-demographic datasets with the highest available granularity to predict the spread of COVID-19 in the province of Barcelona. The model is flexible enough to incorporate the effect of containment policies, such as lockdowns or the use of protective masks, and can be easily adapted to future epidemics. We follow a stochastic approach that combines a compartmental model with detailed individual microdata from the population census, including social determinants and age-dependent strata, and time-dependent mobility information. We show that our model reproduces the dynamical features of the disease across two waves and demonstrates its capability to become a powerful tool for simulating epidemic events.

## 1 Introduction

The COVID-19 outbreak struck society like a tsunami, affecting citizens in many aspects, from mental health to economic well-being. Communities all over the world had to modify habits and social contacts, in a common effort of erecting a barricade against the virus. Understanding the virus, its structure and its dynamics, is of paramount importance not only for the development of medical solutions (vaccines and specific pharmacological therapies) but also to define the social strategy of prophylaxis and prevent the expansion of the pathology.

The contagious spread of COVID-19 and the impact of mitigation strategies are currently the subject of many studies, all of them pursuing a better understanding of the variables at stake (it is worth citing the progenitor studies by Imperial College London (Davies et al., 2020; Walker et al., 2020) and, in the particular case of Catalonia, those conducted by the Computational Biology and Complex Systems group at UPC (The Computational Biology and Complex Systems group, 2021; Agència de Qualitat i Avaluació Sanitàries de Catalunya (AQuAS), 2021; Evolution of number of cases and Rt of SARS-Cov2, 2020); for further references see also the review by Estrada (Estrada, 2020)). This is a complex problem because it involves several factors acting simultaneously, with different weights and consequences, and both short- and long-term effects. What we know for certain is that the disease propagates mainly through social contacts (Khataee et al., 2021). It becomes then crucial to understand the pattern of contacts of each individual – and how this is intertwined with demographic and social determinants – to comprehend the natural history of COVID-19, as well as the specific attributes (age, previous medical conditions, etc) that determine the outcome of the infection in each infected person (Burns et al., 2021). The frequencies and types of contacts are influenced by lockdown policies, while the probability to become infected depends on individual protection measures. Additionally, the probability for infected people to be diagnosed depends not only on the severity of the symptoms but also on testing policies in place. Global epidemiological indicators, like the incidence of the disease, hide a complex entanglement of highly diversified social and individual characteristics that perhaps can be ignored in a first approximation, but ends up being indispensable to model the epidemic.

Several approaches can be pursued. Monitoring indicators, like the daily number of positive tests (The Computational Biology and Complex, 2021; Agència de Qualitat i Avaluació Sanitàries de Catalunya (AQuAS), 2021; Evolution of number of cases and Rt of SARS-Cov2, 2020), are useful to track the dynamical evolution of the outbreak in varying conditions and allow simple short-term extrapolations. These results, however, are very sensitive to initial conditions and need a continuous readjustment of inputs to be reliable in the long run. The exponential amplification of small perturbations in the initial data, typical of epidemics, makes the outcome of an outbreak intrinsically unpredictable (Castro et al., 2020). As for similar forecasting models, epidemic models cannot return exact predictions; epidemic models can, at most, analyse the likelihoods of different scenarios (Wilke & Bergstrom, 2020).

In this context, simulation tools turn out to be decisive to explore *in silico* the phase space of all the known variables (Kerr et al., 2021) and to unravel those steps in the contagion whose interdiction would prevent the epidemic progression (for example, by government policies regarding the introduction of tracking software, quarantine of exposed individuals, selective lockdown or mandatory use of protective masks in certain conditions). The purpose of such tools is thus threefold: first, they are *monitoring* tools, able to catch the evolution of the disease; second, they are *forecasting* tools, used to identify the conditions that could favour an outbreak; finally, they are *prevention* tools that can provide health authorities with studies on the effectiveness of containment measures. Analytical models as simulation tools usually treat the population as an aggregated body without taking into account its spatial variability or its social and demographic diversity. Some relevant features of COVID-19 can still be inferred from more sophisticated mathematical models (for Catalonia, for example, this has been done following a microscopic Markov chain approach applied to an age-stratified meta-population model (Arenas et al., 2020)). The loss of granularity, however, precludes the design of fine-tuned policies, which in turn renders the decision-making process not fully adequate in all aspects and for all groups, as proved not long ago for the case of H5N1 influenza (Ferguson et al., 2005).

In this work, we present a different approach, a simulation tool that follows the daily contact network history of each individual in a largely populated area, the province of Barcelona. A precise knowledge of the morphology of these networks has been shown to be of the greatest importance to capture the salient characteristics of the outbreak (Chung & Chew, 2021; Small & Cavanagh, 2020; Wang et al., 2022). For each individual, we consider their corresponding microenvironment: home location and the co-residence structure, the employment situation and the mobility routine with its resulting pattern of contacts. Using such information, we have developed a stochastic compartmental model that includes both population and epidemiological data. On the one hand, population data consist of all information about contact networks and mixing patterns (the “where” and “how” people live and move), based on the following external inputs.

i Socio-demographic individual microdata from the latest census data provided by the Spanish National Statistics Institute (Instituto Nacional de Estadística (INE), 2011).
ii Contacts (country-dependent) matrices for densely populated environments (Enquesta de Mobilitat en Dia Feiner, 2019; Moreno et al., 2021; Prem et al., 2020).
iii Mobility data, reconstructed from information supplied by mobile network operators (Instituto Nacional de Estadística (INE), 2020a; Instituto Nacional de Estadística (INE), 2021).

On the other hand, epidemiological data (Burns et al., 2021) contain information related to the disease itself, its dynamics, and the different phases of an infectious process (exposure to the virus, infection, diagnosis and recovery). In this regard, we use a compartmental model, where the entire population is classified into five states: “susceptible”, “exposed”, “infected”, “diagnosed” and “recovered” (see Sec. 3).

Our simulation tool calculates the daily probabilities for each individual of becoming infected at home, at work or school, during further social contacts (generically labelled as “Community”) and in public transport. Age, co-residence patterns, work activity, local mobility and the usage of public transportation are used to estimate the contacts network of each individual. Measures modifying the network and the frequency of the contacts (lockdown, teleworking, etc) are also taken into account, as well as the introduction of protective masks that modify viral load transmission. The tool is configured with a total of 64 parameters to describe the disease, the contacts and the protection measures. Most of the parameters (56) are set a priori to default values (see Supplementary Material), while 8 are suitably calibrated with data (see Sec. 3).

## 2 The COVID-19 pandemic in the province of Barcelona

Our goal is to simulate the COVID-19 outbreak within the population of the province of Barcelona (5.5 million inhabitants) and reproduce the first two epidemic waves experienced in the region during February-June and July-December 2020 for a total of 300 days. We assume the existence of an initial set of about 50 exposed people (0.001% of the population), randomly distributed across the region. The actual location of people changes throughout the day and throughout the week: the weekdays of workers and students, for example, are divided into three 8-h fractions according to a given pattern (home ⇒ job (or school) ⇒ other activities). The pattern of allowed locations and activities evolves according to lockdown and mobility restrictions promulgated during such 300 days.

**Fig. 1:**
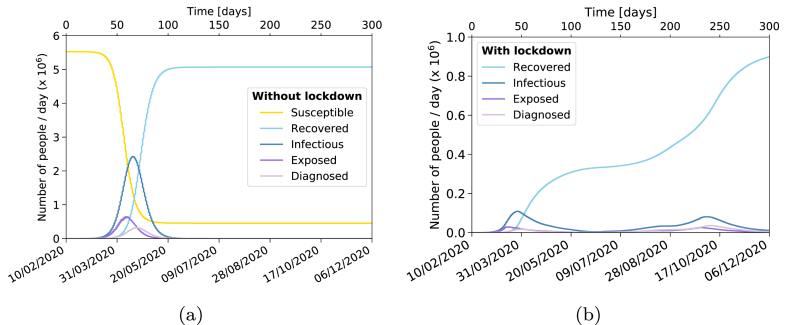
**Evolution of the COVID-19 pandemic in the province of Barcelona.** Number of susceptible, exposed, infected, diagnosed and recovered people as a function of time over a period of 300 days from February 10 to December 6, 2020. It has been assumed that about 50 people, chosen at random, are exposed at time zero. **a** No lockdown measures. **b** Lockdown measures are applied (the susceptible compartment is omitted to ease the visualisation of the information from the other compartments).

Our tool predicts the change of the epidemiological status of every individual at each time interval: becoming exposed as a result of their contacts, becoming infectious if already exposed, being diagnosed, or recovering. We can thus track the number of individuals in each epidemiological compartment as a function of time, as shown in Fig. 1. Without including the impact of lockdown measures in the simulation (Fig. 1a), more than 90% of the total population of Barcelona and its province get affected by the disease in about 100 days. When including them (Fig. 1b), the amount of infected people reduces to 300k in about the same period. In the second wave, continuing until the end of the year, the total number of infected people increases to 850k.

**Fig. 2:**
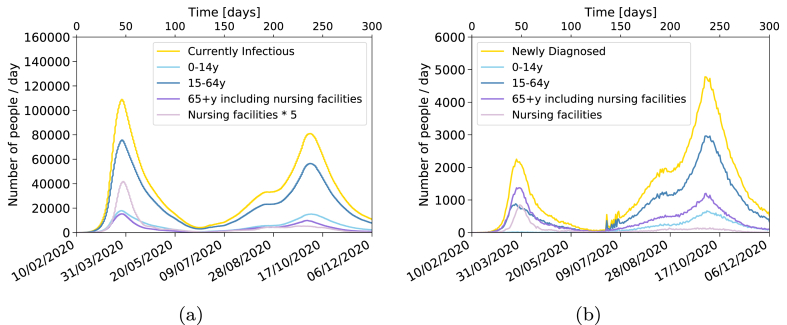
**Time evolution of infected people.** The number of infected people over a period of 300 days is shown split by age categories: less than 15 years old, from 15 to 64 years old and 65 years old and above (hosts in nursing facilities are shown separately). It has been assumed that about 50 people, chosen at random, are exposed at time zero. **a** Total number of infected people in a given day. The number of people in nursing facilities is multiplied by five to make it more visible. **b** Newly diagnosed people every day. In this case, the number of people in nursing facilities is not multiplied by five.

Fig. 2 presents the numbers of currently infected and newly diagnosed people predicted by the simulation as a function of time. The figure presents cumulative and age-dependent evolution curves. The subset of hosts of collective accommodation institutions (nursing homes) is shown separately. The cumulative curve shows a first wave characterised by a steep onset, interrupted by the strict lockdown measures established on March 16, 2020. The mobility restrictions induce the turn-over of the curve and its subsequent decay over a time scale related to the duration of the incubation and infectious phases of the disease. Contacts start increasing again during summer, leading to a second wave in autumn. Adults constitute the most relevant fraction of the infected compartment during both waves. The category of diagnosed is instead dominated by seniors in the first wave and adults in the second.

**Fig. 3:**
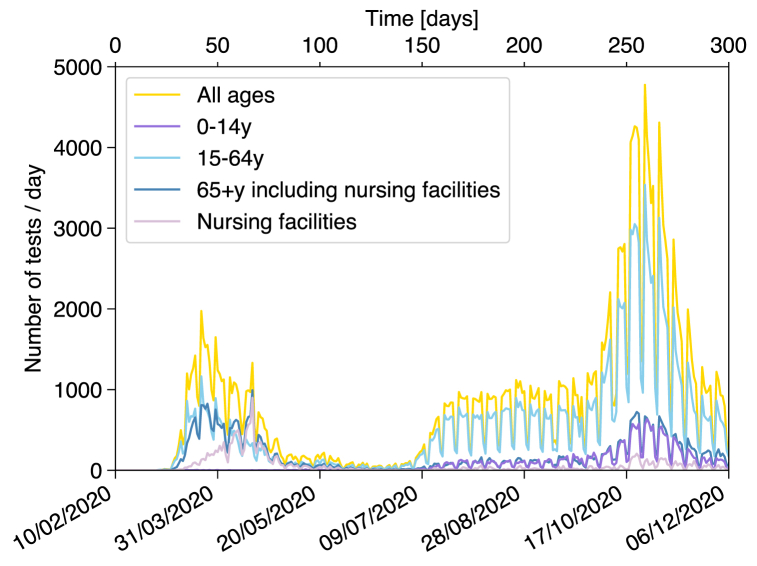
**Number of positive PCR tests in the province of Barcelona.** The number of positive PCR tests in the province of Barcelona for the period between February 10 and December 6, 2020 is shown separately for the different age segments of the population, including its sum.
ii We analysed under which circumstances people become infected either being at home, at work or school, or involved in social activities, such as shopping, leisure time, etc. Public transportation effects are taken into account: mostly during work time for those using mass transportation for their route to work, or during the weekends or the week for those citizens using group travel systems for other activities (see Sec. 3). In our simulation, the amount of available environmental virus, or the viral load each individual is exposed to, is encoded in the *force of infection* *λ*, an aggregate parameter accounting for the probability to change the status of an individual from susceptible to exposed (see Equation (1) in Sec. 3). The results in terms of the viral load are shown in Fig. 4. This is an effective way to forecast the risk of contagion in different social activities. The figure shows that in the early days before the lockdown, the viral load exposition is roughly equal at home and at work or school, being slightly higher for community activities. However, when strong lockdown measures are applied on March 16, the contributions from work and community drop drastically and contagion at home becomes the most relevant contribution. The exposition to viral load in public transport or due to casual contacts during summer activities is relatively small, overall of the order of 1%, but is nevertheless a relevant component as it can bring the virus to households and work communities that would otherwise not be exposed to the infection.

Relevant results have been obtained by considering separately age-, social-activity- and region-dependence.

i The number of diagnosed people, as shown in Fig. 2b, can be compared with data provided by the Catalan Health Service (Agència de Qualitat i Avaluació Sanitàries de Catalunya (AQuAS), 2021), as shown in Fig. 3. The outcome of the simulation resembles quite closely the real picture that emerged in these months for the number of positive PCR tests. Seniors in nursing facilities are notably more numerous than expected from their natural demographic fraction. This pattern results from the joint action of three factors. First, the probability of developing a high viral load with strong symptoms increases significantly as a function of age starting at 65 years-old, while adults have a much lower probability to develop strong symptoms, and even less so children. Second, tightly knitted communities as those of nursing facilities favour multiple contacts. Third, during the first wave, people with strong symptoms would reach the public health system faster and have a higher probability of being diagnosed. The features of the second wave with a peak in October are also qualitatively reproduced, with the spread of the disease picking up again during summer. In this case, adults form the group dominating the diagnosed compartment, with children further contributing visibly. The special protection measures taken in nursing facilities (wearing protective masks) reduced contagion, while the number of tests (and hence the probability to get diagnosed) increased and reached a larger part of the population. Our simulation shows that the unusual strength of the second wave has its root in extra occasional contacts taking place during the summer.

**Fig. 4:**
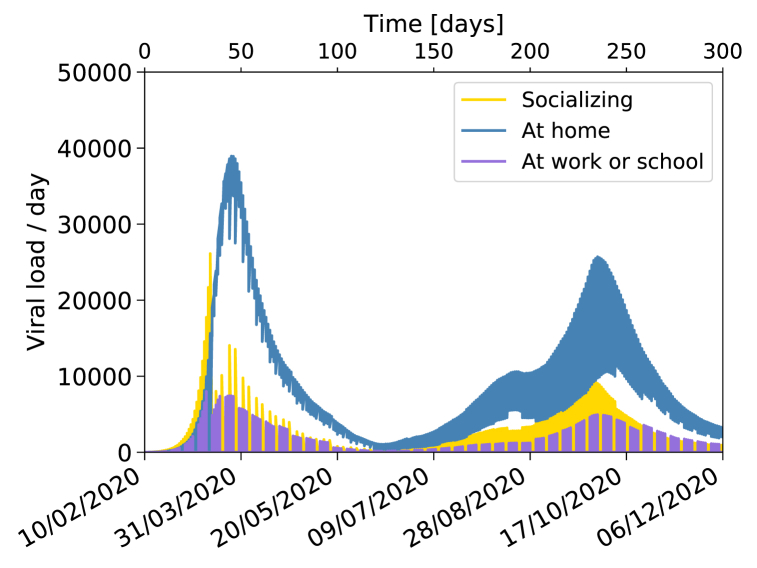
**Total viral load evolution.** The evolution of the main components of the viral load to which the population is exposed is shown separately for time spent at home, at work or school, or in social/community interaction. It has been assumed that about 50 people, chosen at random, are exposed on February 10. The time evolution is shown for 300 days from February 10 to December 6, 2020.
iii We investigate the spatial dependence within our simulation. Fig. 5 shows a comparison between real data (Agència de Qualitat i Avaluació Sanitàries de Catalunya (AQuAS), 2021) and the corresponding simulations in three (sequentially concentric) regions centered on the city of Barcelona. (see Supplementary Material for a map): the Barcelonès – 2.3 million inhabitants –, a first “ring” – 2.5 million inhabitants – and a second “ring” – 0.8 million inhabitants.

**Fig. 5:**
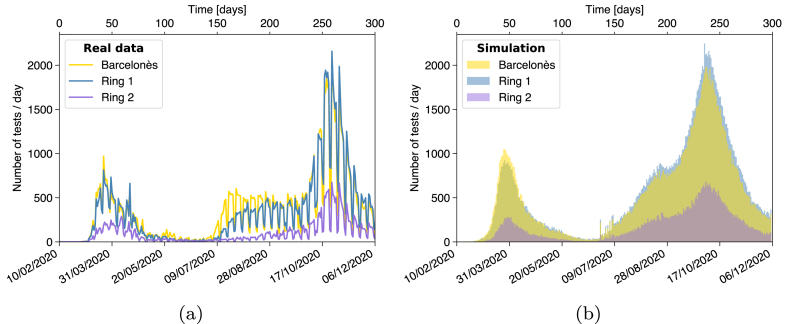
**Number of positive PCR tests in the province of Barcelona.** The results are shown separately for the Barcelonès county, and the counties surrounding it for the first and the second ring. **a** Data. **b** Simulation.

The characteristics of the waves and the level of agreement between data and simulation are different for the first wave during the spring and the second wave during summer-autumn. There is good agreement in the intensity and shape of the first wave. The Barcelonès and the first ring have a faster rise interrupted by the lockdown measures, while the disease propagated more slowly in the outer ring, less urban and densely populated. On the other hand, the data of the second wave are quite distinct. The Barcelonès exhibits a sharper increase in the number of diagnosed people in early July, followed by a plateau until a clear peak develops. Data in the first ring grow slower but also reach a plateau before the appearance of the second peak, while data in the second ring rise slowly until the peak develops. We observe in the simulation that additional contacts in the summer are necessary to explain the appearance of a second wave, which the limitation of mobility and mask protection would otherwise prevent. The simulation is able to reproduce the relative size of the waves in the three regions, but not the summer plateau. If we inspect the data with more spatial granularity, we realise that this apparent plateau is an artefact resulting from to the superposition of shorter waves occurring at different times in different sub-regions. Some of them can be correlated to specific local events. These features are currently not being captured by the simulation in all their details, but surely deserve more attention in future studies (see Sec. 3 for further discussion). As a consequence, the summer waves in the different regions are more in phase and appear more like a shoulder to the left of the peak. This also influences the time when the second wave develops, about two weeks earlier in the simulation than in the data.

## 3 Mathematical model, data analysis and calibration

The simulation described in this work reproduces the very different characteristics of the first and second wave of the COVID-19 outbreak in the province of Barcelona on the basis of both epidemiological data and population statistics, linking the “who”, “where”, “how” and “when” with the attributes of the virus itself. In this section, we detail the different aspects of the calculation. In particular, we discuss: the underlying compartmental model; the data used to characterise the disease and the population (with particular emphasis on the issue of nursing facilities); the description of contacts between individuals; how mobility data are processed and used to simulate the impact of lockdown measures; the implementation of other mitigation strategies (such as the use of protective masks); the way this information is processed in order to produce the total force of infection; and finally, how the model is calibrated with the data.

### 3.1 SEIDR model

We consider an agent-based model where the population is divided into five compartments: “susceptible”, “exposed”, “infected”, “diagnosed” and “recovered” (note that in our model we do not include traditional vital dynamics: births and deaths). When susceptible individuals come in contact with infected persons, their state may change to exposed according to the probability

(1) $$P=1-e^{-\lambda_{i}\cdot \Delta t}$$

where the force of infection *λ*_*i*_ is the total viral load an individual *i* is exposed to per unit time (day) and Δ*t* is the time interval (1/3 day).

**Fig. 6:**
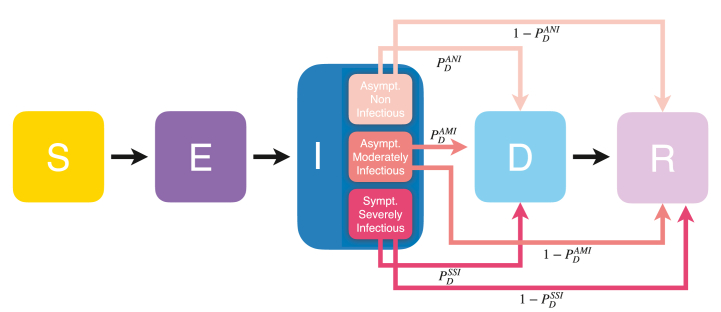
**Transition diagram for our SEIDR model.** When becoming infected, individuals are assigned a sub-compartment of disease characteristics with different symptomatology and infectiousness according to their age strata (0-14y; 15-64y; 65 + y): asymptomatic non infectious (ANI), asymptomatic moderately infectious (AMI) and symptomatic strongly infectious (SSI). In turn, individuals are assigned the specific probability of being diagnosed of their sub-compartment (*P*_*D*_) and conversely of transiting directly to the recovered compartment 1 − *P*_*D*_.

Once exposed every person will transit through the subsequent states until recovery, as shown schematically in Fig. 6. The timeline of the transitions is the result of the specific disease evolution of the individual concerned. The incubation time governs the transition to “infected” and is randomly attributed according to a probability distribution. Other characteristics include the intensity of virus shedding and of symptoms, which are in turn related to the probability of being diagnosed. Not all infected people will be diagnosed, and some will transit directly from “infected” to “recovered”. The time to recovery is fixed. Recovered people are immune and do not become susceptible again.

In the next paragraphs, we describe in detail the characterisation of the disease, the population with their activities and contacts, the lockdown and protection measures, how this information is combined to calculate the force of infection *λ*_*i*_ and the timeline of the transitions, as well as the calibration procedure.

### 3.2 Characterisation of the disease

If exposed, individuals are assigned a given set of variables to describe the evolution of the disease in their specific case. Every exposed person will eventually transit to the infected state. Epidemiological data are available for the incubation time defined as the time between infection and onset of symptoms (Bi et al., 2020). These data are described by a Gamma distribution with an average of *μ* = 4.58 days and standard deviation *σ* = 3.24 days. The most probable value for incubation time is 3 days and the maximum allocated incubation time is 14 days. Epidemiological data show that the infectiousness process (shedding of virus) starts about two days before the onset of symptoms (He et al., 2020). The beginning of that process defines the transition from the susceptible to the infected state in the simulation and the beginning of the viral load shedding. In the simulation, the time-dependent profile of viral shedding *F*_TimeProfile_ is described by a Gamma function with average *μ* = 2.5 days and *σ* = 1.7 days. The maximum emission takes place after about 1.3 days. This means that people shed a significant part of their total emission of viral load before the appearance of symptoms.

Further characteristics of the disease are set in the simulation according to the results of studies of epidemiological data from the beginning of the epidemics (Aleta et al., 2020; Análisis de los casos de COVID-19, 2020; Bi et al., 2020; Di Domenico et al., 2020; He et al., 2020; Soriano-Arandes et al., 2021; Verity et al., 2020). Each member of the infected compartment is classified according to two aspects of the disease: the existence of symptoms (“symptomatic” or “asymptomatic”) and the amount of viral shedding (“strongly infectious”, “moderately infectious” or “non-infectious”). Depending on the context, either one aspect or the other is most relevant. We have defined three relevant combinations: asymptomatic non-infectious (ANI), asymptomatic moderately infectious (AMI) and symptomatic strongly infectious (SSI), as shown in Fig. 6. The probability of belonging to such classes is strongly age-dependent: older people have a higher probability to develop symptoms and be more infectious, while children are more likely to be ANI. In addition, we assume a relation between the intensity of symptoms and infectiousness: infectious people with symptoms emit about twice as much viral load as asymptomatic infectious people. The overall level of intensity of infectiousness *I*_Infectiousness_ is set for every individual according to these categories. Furthermore, to calculate the number of diagnosed individuals, we have to take into account the probability of being diagnosed for each category. During the first wave, PCR tests were done mainly for people with strong symptoms, while during the second wave they would include close contacts of identified cases and random mass screenings. This is reflected in the setting of probabilities of detection. In the first wave $P_{D}^{\mathrm{SSI}}$ = 1, while $P_{D}^{\mathrm{ANI}}$ and $P_{D}^{\mathrm{AMI}}$ = 0. In the second wave $P_{D}^{\mathrm{SSI}}$ = 1, while $P_{D}^{\mathrm{ANI}}$ = 0.4 and $P_{D}^{\mathrm{AMI}}$ = 0.5. Correspondingly, the probability to be diagnosed for the age strata 65 + y is 70% in the first wave and goes up to 85% in the second wave, for the age strata 15-64y the probability changes from 15% to 55%, and for the age strata 0-14y from 1% up to 30%. In the simulation, the transition takes place in eight steps between June 25 and July 7, causing some discontinuities in the daily number of newly diagnosed people as seen in Fig. 2b. People diagnosed are informed of the result after a time calculated according to a Poisson distribution with an average of six days since the start of infectiousness, a minimum of three and a maximum of 14 days (Análisis de los casos de COVID-19, 2020). In the simulation diagnosed individuals are immediately put in effective quarantine and are thus no more contagious. All infected and diagnosed people will eventually move to the recovered state, at the latest 14 days after the start of infectiousness (Tolossa et al., 2021). Hospitalisation, admission to intensive care and possible death have not been implemented so far. Recovered people are considered immune.

### 3.3 Population data

To simulate the history of each of the 5.5 million citizens of the Catalan province of Barcelona, we create a directory, based on the most updated census (“Cens de població i habitatges”) (Instituto Nacional de Estadística (INE), 2011), with an entry for every individual. The original data set consists of approximately 400,000 people, each carrying an effective weight to account for the whole population. Each entry is characterised by a household identifier, age, gender and further relevant characteristics (e.g., occupation, location of the workplace, commuting time - if any -, commuting means of transport - if any -, etc.). The province of Barcelona is divided into 83 areas: notably, the city of Barcelona is divided into 51 neighbourhoods, while the rest of the province is split into 32 regions distributed in concentric areas around Barcelona city (see Supplementary Material).

### 3.4 Nursing facilities

During the first months of the health emergency, the COVID-19 disease caused a high number of deaths among the elderly. In particular, the long-term care facilities suffered from unfortunate notoriety due to the high mortality rate of their residents compared to people of the same age group living in family dwellings. The census we employed to reconstruct the population does not take into account people aged 65 years or over who reside in nursing facilities. In order to include this sector of the population, we used the list of the 756 official long-term care facilities established in 2019, along with details regarding the available places therein (Centro de Ciencias Humanas y Sociales CSIC, 2019). The relative age structure of the elderly population living in these facilities is almost symmetrically inverse to that of the elderly population living in family dwellings (Instituto Nacional de Estadística (INE), 2020b): the oldest among the elderly live mainly in institutions, while the youngest live in private homes. Given the average occupancy level of nursing facilities at 86% (Diputació de Barcelona, 2020) and the location of each of the facilities, we reconstruct the profile for the estimated population of 40,000 individuals hosted in nursing homes. The ages of each of the individuals are randomly assigned according to the overall age structure.

### 3.5 Description of activities and contacts

The history of activities of every individual is simulated as a function of time. The time is organised in weeks, with five labour days and two weekend days. A day is subdivided into three 8-h intervals. The three daily time intervals correspond to time spent: i) at home, ii) at work/school for workers and pupils (or at home for the rest), and iii) in generic *social activities*. During the weekend, people are either at home or involved in social activities. The weekly pattern of occupations, together with the granularity of 8-h intervals, is customisable. This pattern is modified with time according to lockdown measures (e.g., closure of schools or promotion of teleworking). We define five categories of contacts.

i Home: The home contacts include the explicit group of individuals sharing the same household or nursing facility. The average number of home contacts per person is 2.73.
ii Work and School: We assume closed groups whose sizes follow a Poisson distribution with a mean of 10 for work, and fixed sizes for school classes varying from seven for 0-year-old to 28 above 12-year-old (Departament d’Ensenyament, 2020). In the simulation, all workers and students are assigned a company or a school class, which is constant for the whole simulation period. Contacts happening in these reduced groups are stable and long-lasting. In both cases, we use the census information on both the proximity of the workplace and commuting time to simulate a geographical distribution of companies and schools. There are 2.2M workers and 950k school pupils. On average, the resulting number of contacts per person at work or at school is 13.6.
iii Community: Here we include social contacts (other than in the family or occupational bubbles) that we generically label as “Community” contacts. The number of assigned contacts is distributed as a Gaussian with means taken from the largest and most up-to-date synthetic contact matrices set (Prem et al., 2020). The average number of contacts is age-dependent, but not the age profile of these contacts. Contacts are randomly assigned within the same region and are again assumed to be stable contacts but not of the “bubble” type: indeed, each element *A* has its own fixed “Community” network C (the “friends circle of *A*”), however, an element B∈C has a “Community” network $C^{\prime }$ that does not necessarily coincide with that of *A*. On average, such contacts sum up to 4.8 per person.
iv Public transport: A fraction of people use public transportation to commute and a further fraction for community activities. This may lead to additional occasional contacts beyond the network of stable contacts described above. Using the most recent survey on the use of means of transport in the province of Barcelona (Enquesta de Mobilitat en Dia Feiner, 2019), we estimate that 20% of schoolchildren plus 30% of workers use public transport on their way to school and workplace. We further estimate that 10% of the total population uses public transport for other personal reasons. We assume an effective number of about one occasional such contact per round trip and that the users all carry the average viral load of the population using public transport to go to work/school, or for community activities, depending on the case.
v Occasional contacts during community activities: There may be further contacts of occasional nature, similar to the case of public transport. We assume that such contacts took place during summer of 2020 as a result of the relaxation of lockdown measures coinciding with typical summer activities in crowded outdoor settings (traditional festivals, cultural events, beach tourism). Some of these activities included also the additional presence of travellers from outside the province, modifying the effective population (Departament d’Estadística i Difusió de Dades, 2021). In the simulation, the number of such contacts is set to one per person with people carrying a viral load equal to the average of the full population.

### 3.6 Mobility data

**Fig. 7:**
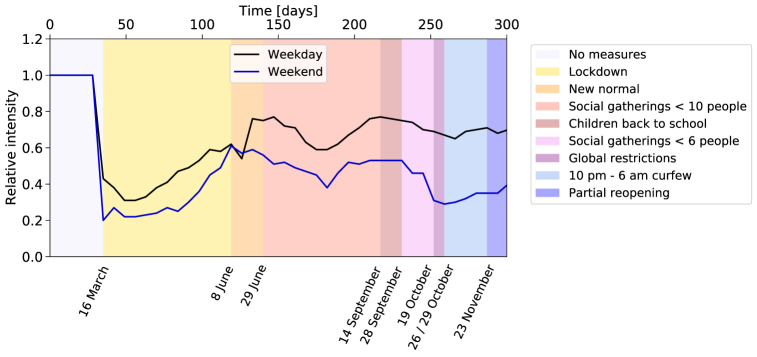
**Relative intensity of mobile phones traffic and measures affecting work and community activities.** Ratio between the intensity of mobile phones traffic during the period covered by the simulation, and for a reference week in November 2019. The pattern is influenced by the several containment measures adopted throughout the year. On March 16, 2020, the Spanish government decreed a state of alarm imposing a succession of lockdown measures starting on the same day and lasting until June 8. The number of people in gatherings was limited to 10 and 6 persons on June 29 and September 26, respectively. Teleworking was encouraged, shops' and cultural events' capacity was reduced to 30% and 50%, respectively. Among other containment measures enforced on October 19, restaurants were closed, and university lectures made virtual.

To control the spread of the disease, authorities imposed lockdown measures restricting mobility, starting from March 16, 2020, and lasting for the rest of the year. We use the mobility data provided by the Instituto Nacional de Estadística (INE), extracted from the analysis of the position of more than 80% of the mobile phones by the three main Spanish mobile phone operators (Instituto Nacional de Estadística (INE), 2020a). Only phones with Spanish numbers are considered in the study. The INE aggregated these data into subgroups of the population that remain, enter or leave a set of “mobility areas” (Instituto Nacional de Estadística (INE), 2021). Data are provided for the year 2020, starting from March 16, both for weekdays and weekends. They are normalised to reference data from November 2019. We use the average data of the full Barcelona province as an indicator of work mobility and leisure mobility. The normalised intensity of mobile phones traffic is shown in Fig. 7.

### 3.7 Lockdown measures

To simulate the impact of the lockdown measures (Diari Oficial de la Generalitat de Catalunya, 2020), we combine two methods. Measures like school closure are directly implemented by changing the configuration of the activity in the time intervals. For example, pupils stay at home instead of going to school, and do not use public transport. For the reduction of work or community contacts, we rely on the time evolution of mobility, as justified by the correlation between mobility changes and confinement measures observed in Fig. 7. There is a substantial dip in mid-March when the first lockdown was implemented, after which the mobility slowly increases. A smaller effect is observed around mid-August (possibly related to holidays), and a more pronounced one later towards the end of October associated with a tightening of the measures. Such correlation was also observed in other regions across Europe (Santamaria et al., 2020). In the simulation, we use the reduction of mobility to calculate the fraction of citizens teleworking or staying at home instead of participating in social activities.

### 3.8 Further mitigation strategies

The use of protective masks was initially deterred (mostly due to the attempt of diverting the available masks to the healthcare providers), later encouraged and finally imposed. Studies show that wearing masks reduces virus transmission, although the extent of such reduction is difficult to quantify precisely (Cheng et al., 2021; Wang et al., 2021). The effect of wearing a mask, *F*_Mask_, is implemented by introducing a factor reducing the viral load transmission at work and in community activities and set to roughly estimated values, 0.7 when it was encouraged and 0.35 when imposed. We assume that people in nursing homes started wearing masks inside the facilities as a measure of precaution. We consider that part of the population, mostly young adults, relaxed the mask-wearing discipline towards the end of the year. In the simulation, we limit the reduction of viral load transmission from 0.35 to 0.85 for 13–35 years old people, from the end of October onward by applying a correcting factor *F*_MaskWearing_.

### 3.9 Calculation of the probability of infection

Putting all the above elements together, we can calculate the viral load exposition *λ*_*i*_ of Equation (1) during the three 8-h intervals that an individual spends either at home (Home), at work or school (Work), or in social interactions (Community). In addition, individuals may use Public Transport (PT) to go to work or in the context of social activities (WorkPT or CommunityPT). Additional interactions may take place during the summer in crowded settings (SummerActivities). The force of infection *λ*, where *λ* represents a vector of 5.5M entries, one per individual, is thus expressed as a sum of terms:

(2) $$\lambda \left(t\right)=\lambda_{\text{Home}}\left(t\right)+\lambda_{\text{Work}}\left(t\right)+\lambda_{\text{Community}}\left(t\right)+\lambda_{\text{WorkPT}}\left(t\right)+\lambda_{\text{CommunityPT}}\left(t\right)+\lambda_{\text{SummerActivities }}\left(t\right).$$

Depending on the time interval, one or more terms may contribute. To calculate *λ*, we first have to quantify the viral shedding *κ* of every infectious individual. It results from the product of several factors:

(3) $$\kappa^{i}\left(t\right)=I_{\text{Infectiousness}}^{i}\times F_{\text{TimeProfile}}^{i}\left(t-t_{0}^{i}\right)\times F_{\text{Contagiousness}}\times F_{\text{Mask}}\left(t\right)\times F_{\text{MaskWearing}}^{i}\left(t\right).$$

The first factor $I_{\text{Infectiousness}}^{i}$ represents the overall strength of viral load shedding of an individual, the second $F_{\text{TimeProfile}}^{i}\left(t-t_{0}^{i}\right)$ the relative amount of viral shedding as a function of the time elapsed since the beginning of infectiousness $t_{0}^{i}$, the third *F*_Contagiousness_ an overall calibration factor to convert the viral load into the corresponding force of infection, the fourth *F*_Mask_(*t*) the viral transmission reduction offered by face masks according to the enforcement policy at that time, and the fifth $F_{\text{MaskWearing}}^{i}\left(t\right)$ the effectiveness with which individuals wear their mask. More details about the settings of the parameters are provided in the Supplementary Material. Next, we evaluate the contacts of every individual during that time interval. We distinguish two types of contacts: the ones that take place in networks of individuals, e.g. people sharing the same home, and spurious contacts, e.g. contacts in public transport (see Sec. 3). The first three terms in Equation (2) are of the first type and are described by contact matrices *A* of dimension (5.5M × 5.5M). The vector *λ* results from the product *λ* = *A* × *κ*, where *κ* is a vector of dimension 5.5M. For spurious contacts, we do not identify the specific individuals involved but calculate instead the average value of viral shedding $\bar{\kappa }$ of the group of people potentially involved, e.g. all people using public transport to go to work. The number of contacts is given by a Poisson variation of an estimated mean number of contacts (MC), such that. $\lambda^{i}=Poisson{\left(MC\right)}^{i}\times \bar{\kappa }\cdot$

### 3.10 Calibration procedure

The simulation contains a total of 64 parameters: 26 of which characterise the disease, 32 the contacts and 6 the lockdown and self-protection measures. Many of them have large a priori uncertainties and/or strong time dependence. Validation and calibration with data are thus important. However the number of parameters that can be meaningfully constrained depends on the quality and granularity of the data, and the resulting best-fit values are still subject to systematic uncertainties from the rest of the parameters. We used a simplified fitting procedure in line with the limited constraining power of the available data set at the time. Out of the 64 parameters that control the simulation, 56 were set to default values extracted from sources of information not directly related to the incidence of COVID-19 in Barcelona (see Supplementary Material and references therein). The remaining eight - the parameters most poorly constrained by a priori external knowledge - were calibrated in order to match at best the predicted time evolution of the number of diagnosed people with the one observed in the data.

Different parameters are sensitive to different parts and characteristics of the distribution. The calibration is done in four sequential steps. We perform coarse scans of the parameters under consideration and select the parameter value whose corresponding prediction is closest to the data. No minimisation procedure is applied. First, we need to calibrate the scale factor that converts viral load, calculated in arbitrary units, into the correct force of infection *λ*. This *single* scale factor, *F*_Calibration_ in Equation (3), governs the growth rate of the first wave. Second, we need another *single* parameter, the starting day of the lockdown measures in the simulation. We vary that day in order to approximately reproduce the rate of 2000 diagnosed cases per day observed in the data at the peak of the first wave, as shown in Fig. 3. That day can then be identified as March 15, when the lockdown measures were imposed by the authorities. After adjusting these two parameters, we performed several validation checks. We observe in the simulation that it takes 6 weeks from the initial 50 infections until the start of the lockdown. The initial infections thus took place on February 10. Although the arrival time of the first infectious people in Catalonia is not precisely known, such a date seems reasonable in view of the international context of the development of the disease. We compared the age-dependent distribution and the spatial distribution of the first wave of the simulation with the one in data in the coarse bins available (Fig. 2, Fig. 3 for age-dependence and Fig. 5 for spatial dependence), finding a good qualitative agreement. Third, individuals in each of the three categories of infectiousness need to be assigned a probability to be diagnosed via a PCR test during the first and the second wave. The probability for people with strong symptoms, $P_{D}^{\mathrm{SSI}}$, is high and set to one for both waves. For the categories with milder or no symptoms, the corresponding probabilities $P_{D}^{\mathrm{AMI}}$ and $P_{D}^{\mathrm{ANI}}$ were very small in the first wave but increased significantly during the second wave. These *two* parameters are set to zero for the first wave, while their values are scanned for the second wave and the combination reproducing the observed proportion of diagnosed people by age categories (Fig. 3) is selected. Finally, additional summer contacts are needed in order to increase the risk of regrowth and facilitate the appearance of a second wave. The *four* corresponding parameters (number of contacts, segment of the population affected –the Barcelonès or the full province–, and starting and ending dates) have been scanned. The set of values approximating in the best way the observed proportions of diagnosed people in the three regions (Fig. 5) was selected.

## 4 Sensitivity to parameters

We studied the sensitivity of the simulation to the parameters and identified the most critical ones. This is relevant to understand the weakest points of the mitigation strategies. It also indicates what should be better known for improved precision of the simulation.

First, we report on the impact of parameters governing the response of individuals to the disease.

i If we reduce the overall scale of infectiousness by a factor of two, it takes twice as much time to reach the explosive contagion regime.
ii Reducing by a factor of two the probability to be diagnosed in the asymptomatic non-infectious and asymptomatic infectious categories during the second wave significantly reduces the number of people being diagnosed, and subsequently put in quarantine. As a consequence a factor of 2.5 more people become infected and the number of people diagnosed increases by 30%.
iii The time it takes for people to get diagnosed is drawn from a Poisson distribution with mean six (Análisis de los casos de, 2020). A minimum of three days is set for the notification of the diagnosis. Removing the minimum does not have much impact, but setting it to large values does. If it is set to six days, people are put later in quarantine and more contagions can take place. The number of diagnosed people in the second wave doubles, at the same time the delay in the notification delays the peak by about a week.
iv The effectiveness of the mask is important in the surge and strength of the second wave. The simulation shows that without that protection a second wave would have quickly developed. Changing the nominal reduction of viral load transmission of 0.35 by ± 0.05 changes the number of diagnosed people by ± 50%. With a much stronger reduction, no second wave would develop.

Next we investigate the sensitivity to the pattern of contacts.

i The size of the contact groups at work is given by a Poisson distribution with a mean of 10. Changing it to 5 (20) leads to a reduction (increase) of 15% of diagnosed people. The impact is smaller in the second wave due to teleworking.
ii In addition to the 950k school pupils there are 95k university students. Since our census data did not provide detailed information for this group and there were essentially no presential classes at the university during 2020 after the start of the first lockdown, we considered that this group neither studied, nor worked. To analyse the impact of this assumption, we aggregated them to the pupils, assuming an average size of closed groups of contacts of 15. They resumed presential classes in mid-September. As a result, the peak of the first wave increased by 25% without readjusting the time of lockdown, while the peak of the second went down by 25%.
iii We studied the impact of making the number of community contacts dependent on the density of the population. We tested a model where the proportionality factor varies from 0.6 to 2, between Berguedà and Barcelonès, respectively, the lowest and highest density regions. The average number of community contacts increases from 4.8 to 7.6. During the first wave, the total number of diagnosed increases by 30%. The overall effect is less noticeable during the second wave, but the number of diagnoses in the Barcelonès starts dominating, unlike what is observed in Fig. 5a. Such a strong density dependence is thus disfavoured.
iv The number of additional summer contacts is much more critical than the contacts in transport as they affect potentially a much more significant fraction of the population. Doubling the default level of one contact generates an earlier wave during the period where a relatively flat distribution is observed, while dividing by two reduces the level of diagnoses in early summer by a factor of six, and no second wave develops. We also tested the impact of limiting the extra contacts to the Barcelonès area rather than the whole province. However, this option does not reproduce well the sharing of diagnosed cases in the different regions (Fig. 5).
v Another factor that may influence the evolution of the disease is the location of the initially infected people. In the default configuration, they are randomly distributed geographically in the province. We kept the same total number but randomly varied their location. In the first wave, this results in a variation of the onset time by ± 2.5 days. If we keep the starting date of lockdown fixed, the maximum of the first wave varies by about ±30%. Indeed the starting time of the lockdown measures is quite critical, since one week delay typically results in a factor of three higher number of diagnosed people per day at the peak. The cases with upward fluctuations in the number of diagnosed people in the second wave are, in general, not correlated with the cases of upward fluctuations in the first wave. Only in about 1% of the cases, when the first wave developed very early, the number of infected people grew by a factor of three before the lockdown took effect (the date was kept fixed). These people eventually became immune, and later, no strong second wave was able to develop.

Finally, we studied variations in the description of mobility.

i To test the relevance of mobility and its correlation with the time dependence of the number of diagnosed people, we artificially introduced an additional four weeks of flat level of mobility in July. This has a strongly correlated effect on the time-dependent pattern of the spread, showing that mobility is a crucial ingredient for describing the evolution of the pandemic.
ii The mobility data came in two sets with different methods and normalisations. Increasing the relative normalisation of the second set (after mid-June) by 10% almost doubles the level of infected people and shifts the peak earlier by one week.

In summary, we have explored part of the phase space of the many parameters playing a role in the expansion of the virus. The most sensitive ones include the effect of the protective masks, the probability of diagnosis, the characteristics of seasonal (summer) contacts and mobility. In some cases, more precise external information can be gathered (e.g. likelihood of diagnostic), or the full granularity of the information could be used, leading to possible improvement (e.g. mobility per region). More complex are the cases of protective masks or summer contacts. Improvements could come from a more detailed description of the parameters together with a comparison with high-granularity data in terms of age and location.

## 5 Discussion and conclusions

The first and second COVID-19 waves in the province of Barcelona are of quite different nature (Arauzo-Carod et al., 2021). The first one is the result of the initially free propagation of the virus, later controlled by the application of strict lockdown measures. Diagnostics were strongly related to the appearance of symptoms. During the second wave, mobility restrictions were alternatively relaxed and tightened depending on the epidemiological situation. The use of masks proved to be essential, as well as the increase in the efficiency of diagnosis of asymptomatic individuals and the corresponding quarantine measures.

The simulation tool presented here is able to reproduce the main features of the first and second waves of the COVID-19 outbreak in Barcelona and its province. The detailed description of two extremely heterogeneous categories of data, the sociodemographic and epidemiological ones, is a crucial ingredient. The sociodemographic individual microdata, convoluted with mobility information, are the base for modelling contacts and are necessary in order to correctly reproduce the expansion of the disease governed by the different components of the parameter *λ* describing the total viral load (Fig. 4). Indeed, since the contribution coming from the viral load components linked to work- and community-activities is drastically reduced as soon as lockdown measures prevail, co-residence contagion becomes the dominant factor, making it very valuable to use real census data, as done in our study. The incubation time, contagiousness period, and age-dependent strength of viral shedding are all important in order to predict the time profile of the viral shedding of an individual and the overall reaction time to lockdown measures.

Many ingredients are intrinsic to the population and do not change as a function of time, while others do vary. An important one is the probability of being diagnosed, which was initially linked to the appearance of strong symptoms, but later extended to people with less or no symptoms due to more awareness, contact-tracing and mass testing campaigns. As a result, the probability of detection in adults and children increased, reversing the proportions of diagnosed people by age-strata. The lockdown measures were continuously adjusted by the authorities to maintain a delicate equilibrium between the contention of the virus and sustaining the economic and educational activities. Mobile phone data, clearly correlated to these measures (see Sec. 3) and available separately for work activities and leisure, are used to predict the time dependence of the level of contacts. The simulation also needs to take into account the increased number of contacts taking place during the summer period in order to generate the conditions for the surge of a second wave.

In conclusion, our simulation tool leads to a deep understanding of COVID-19 dynamics in the province of Barcelona. It is able to reproduce the very different characteristics of the two waves, the underlying dynamics of the second one being much more complex. The stochastic approach allows an easy implementation of the many relevant determinants of the problem with the maximum available granularity, including time dependence. One of its advantages is its capacity to study the relative impact of individual factors and to identify the most critical ones (see Sec. 4). The tool is able to reproduce the most relevant features of the data with most parameters set to default values according to external information and only 8 parameters adjusted with data of diagnosed people. With more granular and complete epidemiological, census and mobility data, the model could be improved to capture more detailed features and be more precisely calibrated. The entire machinery can be easily adapted to study the epidemiological picture of any other region for which individual socio-demographic microdata are available. We can also implement the clinical history of hospitalised patients. In the constant stream of new information about the disease, further data regarding vaccination campaigns, coexistence of different variants of the virus, impact of super-spreaders, effect of local small outbreaks, etc., could be easily implemented without having to change the structure of the simulation.

## Data availability

The census data used to build the complete description of the population of the Barcelona province are available from CED and the Instituto Nacional de Estadística, INE, Spanish National Institute of Statistics: 2011 Population and Housing Censuses.

However, restrictions apply to the availability of these data, which were used under license for the current and previous studies, and are not publicly available. The census data and the derived data describing the entire population of the Barcelona province are however available from the authors upon reasonable request and with permission of CED and INE.

The data describing diagnosed people in 2020 in the province of Barcelona, aggregated by age or by county, are publicly available at the Agència de Qualitat i Avaluació Sanitàries de Catalunya (AQuAS), https://aquas.gencat.cat/ca/actualitat/ultimes-dades-coronavirus/.

The mobility data are publicly available from Instituto Nacional de Estadística (INE), https://www.ine.es/en/experimental/movilidad/experimental_em_en.htm.

## Code availability

The code used in the simulation is available upon request.

## Author contribution

MB led the conception of the project. MB, LLM, MMan, PM, VV contributed to the modelling design. MB, LG, XJ, AL, LLM, IR took care of data preparation. MB, LG, LLM, MMan, PM, AP, IR developed the code. MB, LLM, PM, VV wrote the manuscript. All authors contributed to the discussion and interpretation of the results, revised critically the draft and approved the final version of the manuscript.

## Declaration of competing interest

The authors declare no competing interests.

## References

[bib1] Agència de Qualitat i Avaluació Sanitàries de Catalunya (AQuAS) (2021). https://aquas.gencat.cat/ca/actualitat/ultimes-dades-coronavirus/.

[bib2] Aleta A. (2020). Modelling the impact of testing, contact tracing and household quarantine on second waves of COVID-19. Nat Social Behav.

[bib3] (2020). Análisis de los casos de COVID-19 notificados a la RENAVE hasta el 10 de mayo en España.

[bib4] Arauzo-Carod J.M. (2021). Do local characteristics act in a similar way for the first two waves of COVID-19? Analysis at intraurban level in Barcelona. J Public Health (Oxford).

[bib5] Arenas A. (2020). Modeling the spatiotemporal epidemic spreading of COVID-19 and the impact of mobility and social distancing interventions. Phys. Rev. X.

[bib7] Bi Q. (2020). Epidemiology and transmission of COVID-19 in 391 cases and 1286 of their close contacts in Shenzhen, China: A retrospective cohort study. The Lancet Infectious Diseases.

[bib8] Burns E. (2021). The natural history of symptomatic COVID-19 during the first wave in Catalonia. Nature Communications.

[bib9] Castro M. (2020). The turning point and end of an expanding epidemic cannot be precisely forecast. Proceedings of the National Academy of Sciences.

[bib10] Centro de Ciencias Humanas y Sociales, CSIC (2019). http://envejecimiento.csic.es/documentos/documentos/enred-estadisticasresidencias2019.pdf%20(2019).

[bib11] Cheng Y. (2021). Face masks effectively limit the probability of SARS-CoV-2 transmission. Science.

[bib12] Chung N.N., Chew L.Y. (2021). Modelling Singapore COVID-19 pandemic with a SEIR multiplex network model. Scientific Reports.

[bib13] (2021). The computational Biology and Complex Systems group.

[bib14] Davies N.G. (2020). Effects of non-pharmaceutical interventions on COVID-19 cases, deaths, and demand for hospital services in the UK: A modelling study. The Lancet.

[bib15] Departament d'Ensenyament (2020). https://educacio.gencat.cat/ca/departament/estadistiques/indicadors/sistema-educatiu/escolaritzacio/ratios/.

[bib16] Departament d'Estadística i Difusió de Dades (2021). https://ajuntament.barcelona.cat/estadistica/catala/Estadistiques_per_temes/Turisme_i_promocio_economica/Turisme/Oferta_demanda_hotelera/evo/th07.htm.

[bib17] Di Domenico L. (2020). Impact of lockdown on COVID-19 epidemic in Île-de-France and possible exit strategies. BMC Medicine.

[bib18] Diari Oficial de la Generalitat de Catalunya (2020). https://dogc.gencat.cat/ca/inici/.

[bib6] Diputació de Barcelona (2020). https://www.diba.cat/hg2/presentacioprov.asp?prid=954.

[bib19] Enquesta de Mobilitat en Dia Feiner (2019). https://www.atm.cat/web/es/observatori/encuestas-de-movilidad.php.

[bib20] Estrada E. (2020). Modeling the present, looking at the future. Physics Reports.

[bib21] Evolution of number of cases and Rt of SARS-Cov2 (2020). https://dlscitizens.blob.core.windows.net/rtreports/archived/20201231/CAT/InformeCasosRt_CAT_09.pdf.

[bib22] Ferguson N. (2005). Strategies for containing an emerging influenza pandemic in Southeast Asia. Nature.

[bib23] He X. (2020). Temporal dynamics in viral shedding and transmissibility of COVID-19. Nature Medicine.

[bib24] Instituto Nacional de Estadística (INE) (2011). https://www.ine.es/dyngs/INEbase/en/operacion.htm?c=Estadistica_C&cid=1254736176992&menu=ultiDatos&idp=1254735572981.

[bib25] Instituto Nacional de Estadística (INE) (2020). https://www.ine.es/en/experimental/movilidad/experimental_em_en.htm.

[bib26] Instituto Nacional de Estadística (INE) (2020). http://envejecimientoenred.es/una-estimacion-de-la-poblacion-que-vive-en-residencias-de-mayores/.

[bib27] Instituto Nacional de Estadística (INE) (2021). https://ine.es/index.htm.

[bib28] Kerr C.C. (2021). Covasim: An agent-based model of COVID-19 dynamics and interventions. PLoS Computational Biology.

[bib29] Khataee H. (2021). Effects of social distancing on the spreading of COVID-19 inferred from mobile phone data. Scientific Reports.

[bib30] Moreno T. (2021). Tracing surface and airborne SARS-CoV-2 RNA inside public buses and subway trains. Environment International.

[bib31] Prem K. (2020).

[bib32] Santamaria S. (2020). Measuring the impact of COVID-19 confinement measures on human mobility using mobile positioning data. A European regional analysis. Safety Science.

[bib33] Small M., Cavanagh D. (2020). Modelling strong control measures for epidemic propagation with networks – A COVID-19 case study. IEEE Access.

[bib34] Soriano-Arandes A. (2021). Household severe acute respiratory syndrome coronavirus 2 transmission and children: A network prospective study. Clinical Infectious Diseases.

[bib35] Tolossa T. (2021). Time to recovery from COVID-19 and its predictors among patients admitted to treatment center of Wollega University Referral Hospital (WURH), Western Ethiopia: Survival analysis of retrospective cohort study. PLoS One.

[bib36] Verity R. (2020). Estimates of the severity of coronavirus disease 2019: A model-based analysis. The Lancet Infectious Diseases.

[bib37] Walker P.G. (2020). The impact of COVID-19 and strategies for mitigation and suppression in low- and middle-income countries. Science.

[bib38] Wang Y. (2021). How effective is a mask in preventing COVID-19 infection?. Med Devices Sens.

[bib39] Wang L. (2022). Quantitative analysis of the impact of various urban socioeconomic indicators on search-engine-based estimation of COVID-19 prevalence. Infectious Dis Modell.

[bib40] Wilke C.O., Bergstrom C.T. (2020). Predicting an epidemic trajectory is difficult. Proceedings of the National Academy of Sciences.

