# Supplementary material for: Stochastic simulation of successive waves of COVID-19 in the province of Barcelona
Source: Infect Dis Model. 2022 Dec 27;8(1):145–58. doi: 10.1016/j.idm.2022.12.005 (PMC9792425; doi:10.1016/j.idm.2022.12.005)
Supplement: Multimedia component 1 [file mmc1.pdf]

Supplementary material.

M. Bosman[1*], A. Esteve[2,3], L. Gabbanelli[1], X. Jordan[4], A. López-Gay[5,2], M. Manera[1,6], M. Martínez[1,7], P. Masjuan[8,1], Ll.M. Mir[1], J. Paradells[4,9], A. Pignatelli[1], I. Riu[1], V. Vitagliano[10,1,11]

[1] *Institut de Física d'Altes Energies (IFAE), The Barcelona Institute of Science and Technology, Barcelona, Spain*

[2] *Centre d'Estudis Demogràfics (CED-CERCA), Barcelona, Spain*

[3] *Serra Húnter Fellow, Departament de Ciències Polítiques i Socials, Universitat Pompeu Fabra, Barcelona, Spain*

[4] *i2CAT Foundation, Edifici Nexus (Campus Nord UPC), Barcelona, Spain*

[5] *Departament de Geografia, Universitat Autònoma de Barcelona, Bellaterra, Spain*

[6] *Serra Húnter Fellow, Departament de Física, Universitat Autònoma de Barcelona, Bellaterra, Spain*

[7] *Institució Catalana de Recerca i Estudis Avançats (ICREA), Barcelona, Spain*

[8] *Departament de Física, Universitat Autònoma de Barcelona, Bellaterra, Spain*

[9] *Departament d'Enginyeria Telemàtica, Universitat Politècnica de Catalunya, Barcelona, Spain*

[10] *DIME, University of Genova, Via all'Opera Pia 15, 16145 Genova, Italy*

[11] *Department of Mathematics and Physics, University of Hull, Kingston upon Hull, HU6 7RX, UK*

**Corresponding author: bosman@ifae.es*

Parameters of the model

The model to simulate the spread of Covid-19 counts with a total of 64 parameters to characterize the disease (26), the contacts (32) and the lockdown and self-protection measures (6). Most of them

are set according to external information. Out of the 64 parameters that control the simulation, only 8 are adjusted. Tables 1 to 7 describe the parameters, their settings and related references.

Table 1: Parameters of the model (part I).

Category	Number	Parameters	Source	Adjusted
Disease				
Incubation time (Gamma distribution)	2	μ, σ = 4.58, 3.24 days	Medical data [1, 2, 3]	No
Infectiousness time profile $F_{\text{TimeProfile}}$ (Gamma distribution)	2	μ, σ = 2. 5, 1.7 days	Medical data [1, 2, 3]	No
Onset of infectiousness in relation to onset of symptoms	1	-2 days	Medical data [2]	No
Relative viral strength of asymptomatic, moderate and severely infectiousness	3	$I^i_{\text{Infectiousness}}$ = 0, 1, 2	Medical data [3]	No
Viral strength to probability of infection conversion	1	$F_{\text{Contagiousness}}$ = 4	Adjusted	Shape & size of 1st wave
Fraction in categories of viral strength (age dependent)	2×3	See table 3	Medical data [3]	No
Probability to be diagnosed in first (second) wave	3×2	P_D^{ANI} = 0 (0.4)<br>P_D^{AMI} = 0 (0.5)<br>P_D^{SSI} = 1 (1)	Medical data [4]	Yes for P_D^{ANI} & P_D^{AMI} during 2nd wave
Diagnosis time (Poisson distribution)	3	μ, minimum, maximum = 6, 3, 14 days	Medical data [4]	No
Recovery time	1	14 days	Medical data [5]	No
Initially exposed	1	0.001%	Estimated	No
Contact's model				
Workplace size (Poisson distribution)	1	$\mu = 10$	Estimated	No
Classroom size (fixed, age dependent)	6	See table 4	Departament d'Educació, Gen-Cat [6]	No
Social contacts (Poisson distribution, age dependent)	16	See table 5	Synthetic contact matrices [7]	No
Spurious summer contacts	4	See table 6	Ajuntament de Barcelona [8]	Shape of 2nd wave

Table 2: Parameters of the model (part II).

Category	Number	Parameters	Source	Adjusted
Use of public transportation				
Fraction of workers (Work)	1	30%	Autoritat del Transport Metropolità (ATM) [9]	No
Fraction of pupils (School)	1	20%	ATM [9]	No
Fraction of population (social activities)	1	10%	ATM [9]	No
Average number of contacts per round trip (work/school) (fixed number)	1	1.0	Estimated	No
Average number of contacts per round trip (social activities) (fixed number)	1	1.1	Estimated	No
Confinement				
Date of 1st day of simulation	1	34 days before March 16, 2020 starting date of first confinement	Diari Official de la Generalitat de Catalunya [10]	Shape of 1st wave
Effect of mask	4	See table 7	Medical data [11,12]	No
Inter-calibration of mobile data	1	1	Estudios de movilidad a partir de la telefonía móvil [13]	No

Table 3: Fraction of asymptomatic, moderately and severely infectious people depending on the age.

Age (years)	Asymptomatic	Moderately infectious	Severely infectious
< 15	0.69	0.30	0.01
15 – 64	0.40	0.45	0.15
> 64	0.05	0.25	0.70

Table 4: Number of children in each age group.

Age	Classrooms' size
0	7
1	12
2	16
3 – 5	22
6 – 11	25
12 - 18	28

Table 5: Average number of stable social contacts depending on the age.

Age	Mean
< 5	3.21
5 – 10	3.88
10 – 15	5.27
15 – 20	5.97
20 – 25	5.26
25 – 30	4.33
30 – 35	4.35
35 – 40	5.51
40 – 45	6.48
45 – 50	4.94
50 – 55	5.26
55 – 60	5.26
60 – 65	5.06
65 – 70	4.05
70 – 75	4.89
> 75	2.95

Table 6: Parameters used to model additional summer contacts.

Parameters	Value
Average number of additional contacts	1.0
Starting date	June 8, 2020
Ending date	September 28, 2020
Fraction of population involved	100%

Table 7: The effect of wearing a mask is simulated by introducing a factor F_{Mask} that reduces the viral load transmission at work or school and in community activities. As of October 26, 2020, a relaxation $F_{\text{MaskWearing}}$ of the discipline in the use of the masks by young adults is assumed.

Use of mask	Viral load reduction
Encouraged	$F_{\text{Mask}} = 0.70$
Imposed	$F_{\text{Mask}} = 0.35$
Relaxed use (from 13 to 35 year-old)	$0.35 \times \text{F}_{\text{MaskWearing}} = 0.85$
Starting date of relaxation	October 26, 2020

Statistics of the sample

Tables 8, 9 and 10 present the statistics of different subsets of the population.

Table 8: Number of people in each age category.

Age (years)	Persons
< 15	861,266
15 – 64	3,704,595
> 64	961,372
$\rightarrow$ in nursing homes	39,373
Total	5,527,233

Table 9: Total population classified as people who work, people who go to school and the rest of the population (”Other”).

Occupation	Persons
Workers	2,247,353
Schoolchildren (0 to 18 year-old)	953,019
Other	2,326,861
Total	5,527,233

Table 10: Workplaces model assumed using the information available in the census file.

Work location	Home region	Time to work	Assigned work region	Workers
At home	Any	–	Home region	197,827
Own municipality	BCN	< 20’	Home neighbourhood	190,316
		> 20’	Another BCN neigh.	254,219
	Counties	Any	Home region	448,229
Another municipality	BCN	Any	Counties	128,165
	Counties	< 45’	Home region	659,138
		> 45’	Outside home region	175,293
Several municipalities	BCN	Any	Counties	42,032
	Counties	Any	Home region	144,856
Abroad	Any	Any	Outside home region	7,278

Description of the province of Barcelona

In the simulation, the province of Barcelona is divided into 83 regions: notably, the city of Barcelona is divided into 51 neighbourhoods, while the rest of the province is split into 32 regions. Table 11 shows the number of inhabitants in the 32 regions. These regions are distributed within three concentric areas with the city of Barcelona at the center, as shown in Figure 1: the Barcelonès – the county including the cities of Barcelona, Badalona, L'Hospitalet de Llobregat, Sant Adrià de Besòs and Santa Coloma de Gramanet, for a total of 2.3 million inhabitants –, a first "ring", which includes Baix Llobregat, Vallès Occidental, Vallès Oriental, and Maresme – 2.5 million inhabitants –, and a second "ring", including Garraf, Alt Penedès, Anoia, Bages, Berguedà, and Osona – 0.8 million inhabitants –. Table 12 shows the number of inhabitants in the 51 neighbourhoods of Barcelona, for a total of 1.6 million people.

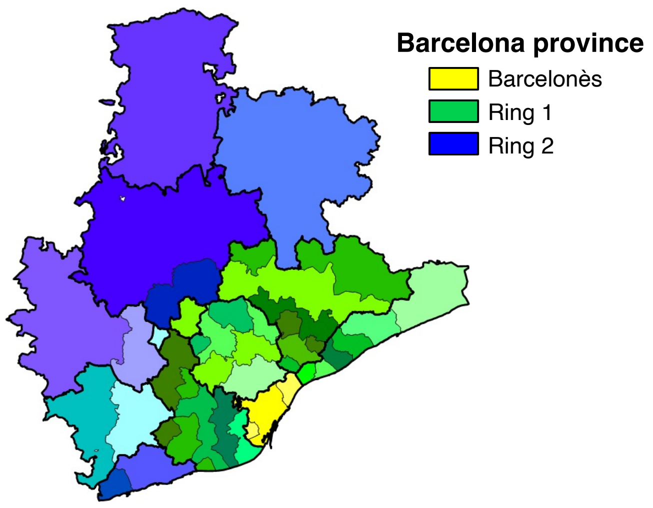

Figure 1: Map of the province of Barcelona. The regions used in the simulation regrouped in three concentric areas.

Table 11: Population in the regions defined in the province of Barcelona census file according to the county they belong to and their distance to the city of Barcelona.

Comarca	Distance (km)	Population
Alt Penedès	30 – 40	72,309
	> 40	25,692
Anoia	30 – 40	29,429
	> 40	88,592
Bages	30 – 40	22,174
	> 40	161,985
Baix Llobregat	< 10	242,777
	10 – 15	253,968
	15 – 20	118,991
	20 – 25	111,045
Baix Llobregat / Alt Penedès	25 – 30	74,370
Barcelonès (excluding the city of Barcelona)	< 10	627,672
Berguedà	> 65	40,292
Garraf	30 – 40	64,702
	> 40	79,947
Maresme	10 – 15	28,159
	15 – 20	66,158
	20 – 25	40,793
	25 – 30	134,370
	30 – 40	46,859
	> 40	115,155
Osona	> 40	151,652
Vallès Occidental	10 – 15	258,591
	15 – 20	344,845
	20 – 25	238,001
	25 – 30	39,125
Vallès Occidental / Oriental	30 – 40	106,489
Vallès Oriental	10 – 15	21,748
	15 – 20	103,441
	20 – 25	25,945
	25 – 30	109,488
	> 40	41,054

Table 12: Population of the neighbourhoods of the city of Barcelona.

Neighbourhoods	Population	Neighbourhoods	Population
Canyelles, les Roquetes	20,748	el Guinardó	35,853
Diagonal i el Front Marítim del Poblenou, Provençals de Poblenou	33,018	el Parc i la Llacuna del Poblenou, la Vila Olímpica del Poblenou	23,576
Hostafrancs, la Bordeta	34,130	el Poble Sec	37,541
Les Corts	46,844	el Poblenou	33,263
Porta	25,265	el Putxet i el Farró	30,337
Sant Andreu (Est)	24,169	el Raval (Nord)	21,234
Sant Andreu (Oest)	34,272	el Raval (Sud)	22,079
Sant Antoni	37,735	el Turó de la Peira, Can Peguera, la Guineueta	34,687
Sant Gervasi-Galvany	43,750	l'Antiga Esquerra de l'Eixample	39,422
Sant Martí de Provençals	25,758	la Barceloneta	17,928
Sant Pere, Santa Caterina i la Ribera	22,348	la Dreta de l'Eixample	41,540
Sants	44,691	la Font d'en Fargues, Horta	36,028
Sants - Badal	24,277	la Marina del Prat Vermell, la Marina del Port, la Font de la Guatlla	41,664
Vallcarca i els Penitents, el Coll, la Salut	36,176	la Maternitat i Sant Ramon, Pedralbes	33,837
Vallvidrera, el Tibidabo i les Planes, Sarrià	27,762	la Nova Esquerra de l'Eixample (Nord)	29,198
Verdun, la Prosperitat	40,707	la Nova Esquerra de l'Eixample (Sud)	32,287
Vilapicina i la Torre Llobeta	26,544	la Sagrada Familia (Nord)	22,534
el Baix Guinardó, Can Baró	36,252	la Sagrada Familia (Sud)	29,771
el Barri Gòtic	17,566	la Sagrera	12,104
el Besòs i el Maresme	19,988	la Teixonera, Sant Genís dels Agudells, Montbau, la Vall d'Hebron, la Clota	29,230
el Camp d'en Grassot i Gràcia Nova	34,582	la Trinitat Nova, Torre Baró, Ciutat Meridiana, Vallbona	18,454
el Camp de l'Arpa del Clot	38,602	la Trinitat Vella, Baró de Viver, el Bon Pastor	21,213
el Carmel	30,183	la Verneda i la Pau	28,426
el Clot	28,550	la vila de Gràcia	49,576
el Congrés i els Indians, Navas	37,642	les Tres Torres, Sant Gervasi - la Bonanova	41,116
el Fort Pienc	30,706		

Age distribution of various sectors of the population

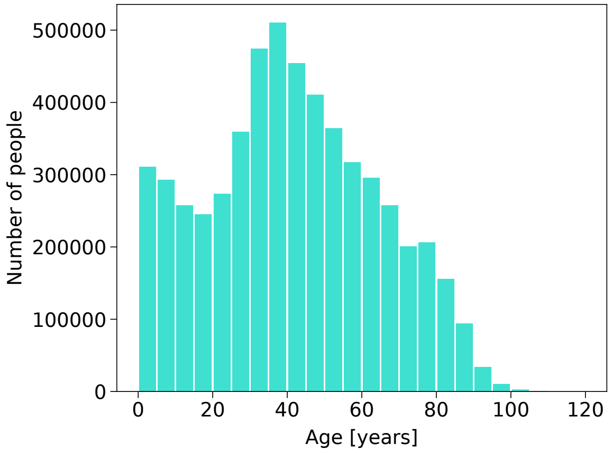

Figure 2: Population age. The age of the population of the province of Barcelona (5.5 million inhabitants) is shown.

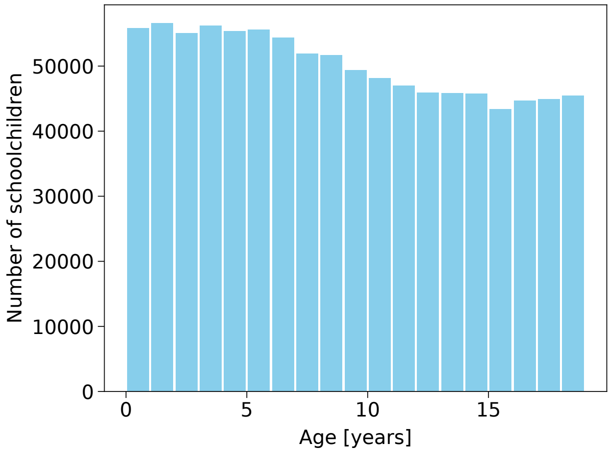

Figure 3: Schoolchildren age. The age of the 0 to 18-year-old children who attend school is shown.

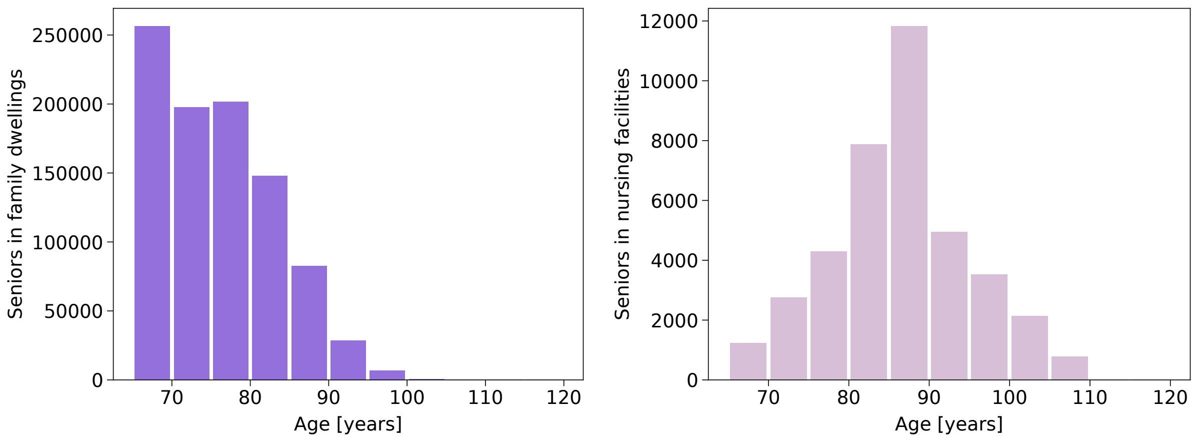

(a) (b)

Figure 4: Seniors age. The age of people over 64 years of age is shown separately for **a** those who live in family dwellings, and **b** those who live in nursing facilities.

Time profile of incubation time and viral shedding

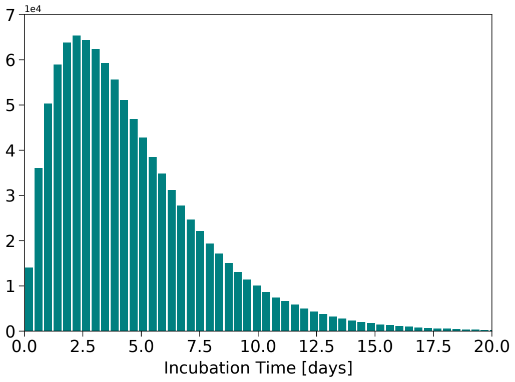

Figure 5: Incubation time. The time of incubation of the virus (in days) follows a Gamma distribution of mean 4.58 days and standard deviation 3.24 days.

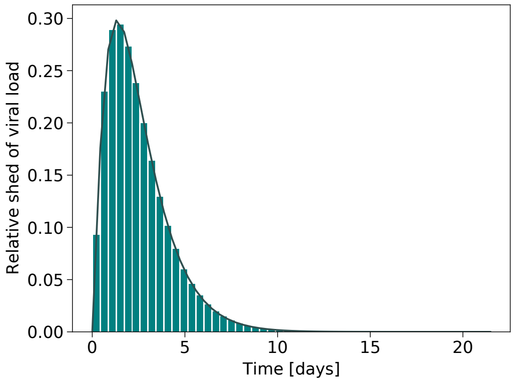

Figure 6: Relative profile of viral shedding. The distribution of the relative profile of the viral shedding follows a Gamma distribution of mean 2.5 days and standard deviation 1.7 days. In the simulation the distribution is truncated at 14 days.

1. Q. Bi et al. Epidemiology and Transmission of COVID-19 in 391 Cases and 1286 of Their Close Contacts in Shenzhen, China: A Retrospective Cohort Study. *The Lancet. Infectious Diseases* **20.8**, 911–919 (2020).

2. X. He et al. Temporal dynamics in viral shedding and transmissibility of COVID-19. *Nature Medicine* **26**, 672–675 (2020).

3. L. Di Domenico et al. Impact of lockdown on COVID-19 epidemic in Île-de-France and possible exit strategies *BMC Med* **18**, 240 (2020).

4. Análisis de los casos de COVID-19 notificados a la RENAVE hasta el 10 de mayo en España. Informe COVID-19 nº 33. 29 de mayo de 2020. https://www.isciii.es/QueHacemos/Servicios/VigilanciaSaludPublicaRENAVE/EnfermedadesTransmisibles/Paginas/-COVID-19.-Informes-previos.aspx (2020).

5. T. Tolossa et al. Time to recovery from COVID-19 and its predictors among patients admitted to treatment center of Wollega University Referral Hospital (WURH), Western Ethiopia: Survival analysis of retrospective cohort study. *PLOS ONE* **16 (6)**, e0252389 (2021).

6. Departament d'Esenyament - Generalitat de Catalunya. Ràtios d'alumnes per estudi i unitat o grup. https://educacio.gencat.cat/ca/departament/estadistiques/indicadors/sistema-educatiu/escolaritzacio/ratios/ (2020).

7. K. Prem et al. Projecting contact matrices in 177 geographical regions: an update and comparison with empirical data for the COVID-19 era. https://doi.org/10.1101/2020.07.22.20159772 (2020).

8. Departament d'Estadística i Difusió de Dades - Ajuntament de Barcelona. Demanda hotelera a Barcelona. Serie històrica 2005-2021. https://ajuntament.barcelona.cat/estadistica/catala/Estadistiques_per_temes/Turisme_i_promocio_economica/Turisme/Oferta_demanda_hotelera/evo/th07.htm (2021).

9. Enquesta de Mobilitat en Dia Feiner (EMEF) - 2019. https://www.atm.cat/web/es/observatori/encuestas-de-movilidad.php (2019).

10. Diari Oficial de la Generalitat de Catalunya . https://dogc.gencat.cat/ca/inici/ (2020).

11. Y. Cheng et al. Face masks effectively limit the probability of SARS-CoV-2 transmission *Science* **372, 6549** 1439-1443 (2021).

12. Y. Wang et al. How effective is a mask in preventing COVID-19 infection? *Medical devices & sensors* **4** e10163 (2021).

13. Estudios de movilidad a partir de la telefonía móvil. https://www.ine.es/experimental/movilidad/experimental_em.htm (2020).
